# Baths and Bathing

**Published:** 1859-05

**Authors:** 


### BATHS AND BATHING.

A cold bath is in water under seventy degrees Fahrenheit, a warm one, is over ninety. A warm bath in pure water debilitates, in salt water, strengthens.

Two cold hip (sitz) baths of fifteen minutes each, during six hours fasting, diminishes the weight of the body one and a half pounds, hence in all appropriate cases is a prompt, efficient, and valuable remedy.

The best, safest, most invigorating bath for persons in health, is to jump into a river of a morning immediately on rising from bed, splurge around for two or three minutes, wipe dry, dress and run home. All who have any regard for personal cleanliness and health, should, once a week, in a warm room, with soap, brush and warm water, give the whole body a thorough washing, then with a cold instantaneous shower bath, or a vessel of water dashed over head and shoulders, wipe, dress, and away.

